# Experimental and clinical evidence of multilayer retinal damage caused by subretinal hemorrhage in neovascular age-related macular degeneration

**DOI:** 10.1038/s41598-026-52680-8

**Published:** 2026-05-13

**Authors:** Akinari Yamamoto, Yuta Nakanishi, Midori Ideyama, Masahiro Akada, Ryosuke Tamiya, Manabu Miyata, Ai Kido, Hiroshi Tamura, Sotaro Ooto, Akitaka Tsujikawa, Masayuki Hata

**Affiliations:** 1 Department of Ophthalmology and Visual Sciences, Kyoto University Graduate School of Medicine, Shogoin Kawahara cho 54, Sakyo-ku, Kyoto City, Kyoto Prefecture 606-8507 Japan; 2 Center for Innovative Research and Education in Data Science, Institute for Liberal Arts and Sciences, Kyoto University, Kyoto, Japan

**Keywords:** Subretinal hemorrhage (SRH), Ganglion cell complex (GCC), Outer nuclear layer (ONL), Neovascular age-related macular degeneration (AMD), Diseases, Medical research, Neuroscience

## Abstract

Neovascular age-related macular degeneration (AMD) with subretinal hemorrhage (SRH) is associated with a poor visual prognosis. This study investigated the effects of SRH in neovascular AMD on visual function and retinal integrity. An SRH mouse model, established by subretinal injection of autologous blood, exhibited early apoptotic cell death across both outer and inner retinal layers, ultimately resulting in retinal ganglion cell loss and inner retinal thinning. Clinically, we retrospectively reviewed consecutive treatment-naïve neovascular AMD patients who visited Kyoto University Hospital between December 2012 and December 2013. Among 43 eyes from 43 patients, the presence of baseline SRH was independently associated with poorer best-corrected visual acuity at 1 year (β = 0.16, *P* = 0.03). Eyes with SRH exhibited significant thinning of the ganglion cell complex (GCC) and outer nuclear layer (ONL). Reductions in GCC and ONL thickness were significantly correlated with worse 1-year BCVA (ρ = 0.51, *P* = 0.02; and ρ = 0.62, *P* = 0.003, respectively). These findings indicate that SRH induces neuronal damage not only in the outer retina adjacent to the hemorrhage but also in the inner retinal layers, both of which contribute to sustained visual impairment in neovascular AMD.

**Supplementary Information:**

The online version contains supplementary material available at 10.1038/s41598-026-52680-8.

## Introduction

Age-related macular degeneration (AMD) is one of the leading causes of blindness worldwide^1^. Among the clinical subtypes of AMD, neovascular AMD is a late stage of the disease with relatively rapid progression and a major cause of severe vision loss^2,3^. Neovascular AMD is characterized by the growth of macular neovascularization (MNV), which can be accompanied by subretinal fluid and/or subretinal hemorrhage (SRH)^4^. In particular, SRH occurs even during intensive intravitreal anti-vascular endothelial growth factor (VEGF) therapy^5,6^. Neovascular AMD with SRH generally has a poor natural course^7,8^ and although several therapeutic approaches for SRH have been reported, visual improvement remains limited^9–12^.

Neovascular AMD complicated by SRH causes substantial damage to the retinal architecture, particularly the outer retinal layers^8^. SRH is the accumulation of blood between the neurosensory retina and the retinal pigment epithelium (RPE)^13^ and the effect of SRH on the neurosensory retina has been experimentally demonstrated. In animal models, SRH has been shown to form clots and fibrin that act as a diffusion barrier, impairing the supply of nutrients and oxygen^14^. This pathological process can also cause mechanical damage such as fibrotic shearing of photoreceptors^15^. Moreover, iron released from hemolyzed red blood cells is thought to promote oxidative stress and inflammatory responses, further contributing to retinal cell damage^16–19^. Finally, eyes with SRH resulted in subretinal fibrosis (38.3%) or atrophic scar (25.0%)^8^.

Although pathological changes in eyes with SRH have been extensively studied, previous studies have mainly focused on the outer retina, and the involvement of inner retina has not been well characterized. A recent study using a SRH model has shown that SRH induces microglial activation in the inner retina, including the ganglion cell layer, suggesting that inner retinal neurons may also be vulnerable^20^. During our preliminary investigation using a mouse model of SRH, we noted unexpected cell death specifically in retinal ganglion cells, raising the possibility that this cell population may also be affected in human disease. Thus, this study aimed to investigate the effects of SRH due to neovascular AMD on visual function and each retinal layer.

## Methods

### Animal experiments

All animal experiments were conducted in accordance with the Association for Research in Vision and Ophthalmology (ARVO) Statement for the Use of Animals in Ophthalmic and Vision Research. All protocols were approved by the Institutional Review Board of the Kyoto University Graduate School of Medicine (MedKyo 23,594). We used adult male C57BL/6JJcl mice (8–10 weeks old; 23–27 g; CLEA, Tokyo, Japan) for histology and in situ TdT-mediated dUTP Nick-end Labeling (TUNEL) assay. B6.Cg-Tg(Thy1-CFP)23Jrs/J mice (12 weeks old; 26–30 g; Jackson Laboratory, Bar Harbor, ME) were used for retinal ganglion cells (RGCs) counting in flat-mounted retinal images. Mice were maintained under a 14-h light/10-h dark cycle with free access to food and water. All efforts were made to minimize animal suffering and reduce the number of animals used.

### SRH mouse model

**Fig. 1:**
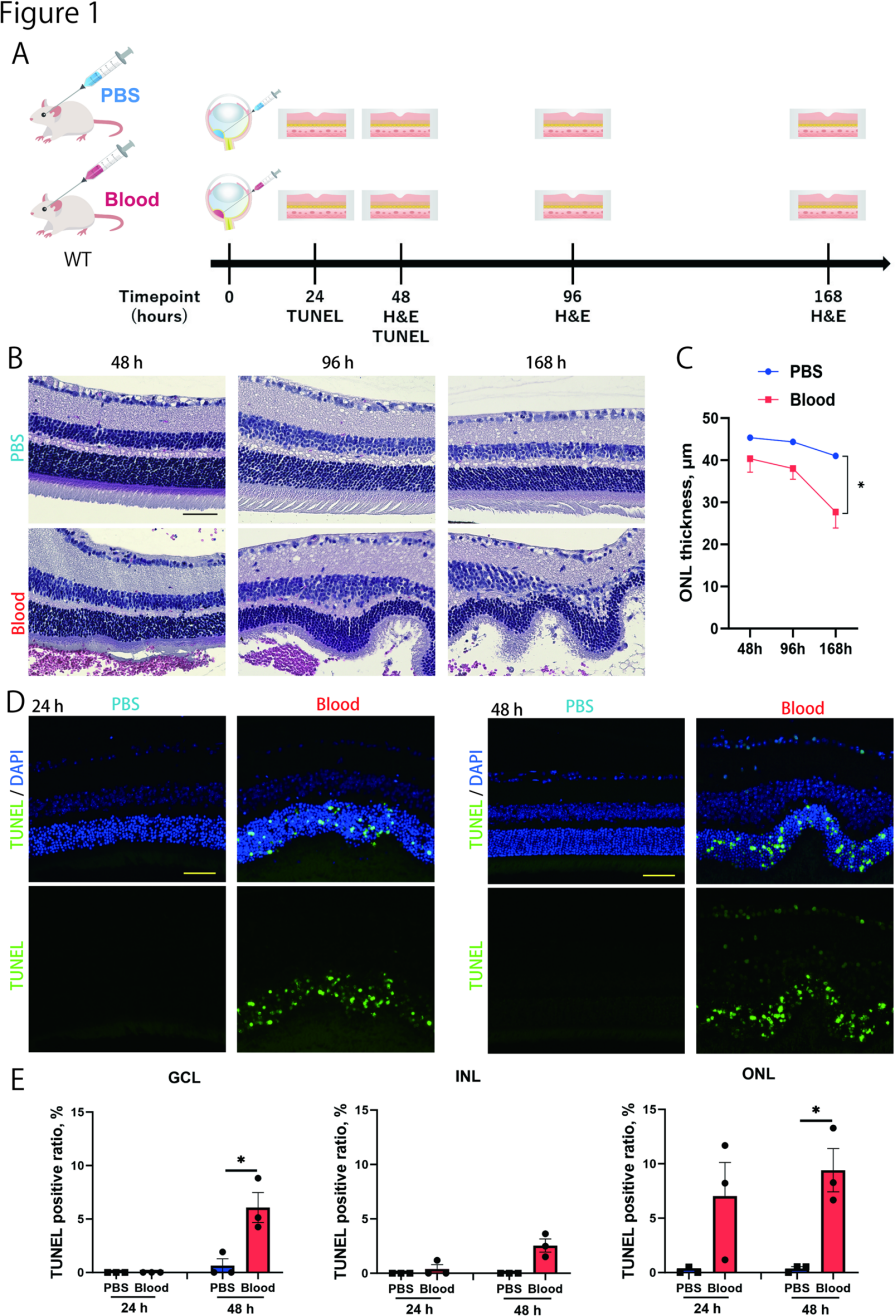
**(A)** Schematic illustration of the experimental timeline. **(B)** Representative hematoxylin and eosin (H&E)-stained retinal sections at 48, 96, and 168 h after blood or phosphate-buffered saline (PBS) injection. Scale bar, 50 µm. **(C)** The graph shows outer nuclear layer (ONL) thickness at each time point. ONL thickness was measured on the retinal pigment epithelium (RPE) at a distance of 500 µm from the optic nerve head (n = 3 eyes from 3 independent mice per group). **(D)** Representative TdT-mediated dUTP nick-end labeling (TUNEL) staining at 24 and 48 h, demonstrating increased apoptotic cells in eyes injected with blood compared with PBS controls. Scale bar, 50 µm. **(E)** Quantification of TUNEL-positive cell ratios in the GCL, INL, and ONL demonstrates significantly higher values in blood-injected eyes than in PBS-injected control eyes (n = 3 eyes from 3 independent mice per group). Unpaired t tests were used (C, E); *P < 0.05; error bars represent mean ± SEM.

Subretinal injections were performed on the side of the optic nerve head, using it as an anatomical landmark, as previously described^21.^ Mice were anesthetized with a mixed anesthetic consisting of medetomidine (0.75 mg/kg), midazolam (4.0 mg/kg), and butorphanol (5.0 mg/kg), administered intraperitoneally (5 μL/g body weight). Pupils were dilated with 0.5% tropicamide and 0.5% phenylephrine hydrochloride eye drops, followed by topical anesthesia with 0.4% oxybuprocaine hydrochloride. For induction of SRH, 1 μL of autologous blood collected from the tail vein was immediately injected into the subretinal space using a 33-gauge needle (Ito Corporation, Shizuoka, Japan). Control eyes received a subretinal injection of 1 μL of phosphate-buffered saline (PBS) using the same procedure (Fig. 1A). For all histological and flat-mount analyses, one eye per mouse was used.

### Histology and retinal section preparation

Eyes were enucleated at the indicated time points after the subretinal injection and fixed in 4% formalin. Paraffin-embedded sections were prepared and stained with hematoxylin and eosin (H&E) or TUNEL. Outer nuclear layer (ONL) thickness was measured at a fixed location 500 µm from the optic nerve head to ensure standardized comparison, corresponding to the region of hemorrhage or retinal detachment.

### In situ TUNEL assay

Retinal cell apoptosis was assessed using a MEBSTAIN Apoptosis TUNEL Kit Direct (MBL, Tokyo, Japan) according to the manufacturer’s instructions. Images were acquired from the central region of SRH or retinal detachment area using a fluorescence microscope (BZ-9000; KEYENCE, Osaka, Japan). The ratio of TUNEL-positive cells to total retinal cells was calculated.

### RGC counting

The number of CFP-positive cells was quantified in retinal flat-mounts. 28 days after the injection of blood or PBS, the eyeballs were removed and the retina was whole mounted and imaged using a fluorescence microscope (BZ-X710; KEYENCE, Osaka, Japan). CFP-positive cells were counted using ImageJ software (version 1.54 g; National Institutes of Health, Bethesda, MD, USA), and cell density (cells/mm^2^) was calculated by normalizing the cell counts to the analyzed retinal area.

### Clinical study design

The ethics committee of Kyoto University Graduate School of Medicine (Kyoto, Japan) approved this retrospective observational study (approval number R0532). All study protocols adhered to the tenets of the Declaration of Helsinki. The participants provided written informed consent prior to enrollment.

### Participants

We recruited consecutive patients with treatment-naïve AMD who visited Kyoto University Hospital between December 2012 and December 2013. The inclusion criteria were as follows: (1) age older than 50 years, (2) axial length less than 26.5 mm, and (3) followed up for at least one year after first injection of aflibercept. The exclusion criteria were as follows: (1) patients who had received treatments other than anti-VEGF treatment (e.g., gas injection, photodynamic therapy or vitrectomy), (2) patients with other retinal diseases such as retinal vein occlusion, diabetic retinopathy or glaucoma, (3) cases with retinal angiomatous proliferation (RAP), and (4) poor image quality for analysis in spectral-domain optical coherence tomography (SD-OCT). Patients who developed new SRH during follow-up were excluded from the analysis when new SRH occurred, and those lost to follow-up after the second year were also excluded from that time onward.

### Intervention and observation procedure

In the first year, all patients received three monthly injections followed by four two-monthly injections of aflibercept. After the first year, additional injections were administered as needed based on AMD activity (pro re nata).

Prior to treatment, all patients underwent a comprehensive examination, including measurement of best-corrected visual acuity (BCVA) with a Landolt chart, measurement of intraocular pressure, slit-lamp biomicroscopy, fundus ophthalmoscopy, measurement of axial length (AL) using partial coherence interferometry (IOL Master 500; Carl Zeiss Meditec, Dublin, California, USA), color fundus photography (TRC-NW8F; Topcon, Tokyo, Japan), SD-OCT (Spectralis; Heidelberg Engineering, Heidelberg, Germany), fluorescein angiography (FA), indocyanine green angiography (ICGA) and fundus autofluorescence imaging (HRA2; Heidelberg Engineering, Heidelberg, Germany). We performed fundus photography, angiography, fundus autofluorescence imaging and SD-OCT under pupillary dilation. The SD-OCT images included 30° horizontal and vertical normal and enhanced depth scanning through the fovea with an average of 100 scans. Volume scans were performed using 13 raster scans with an average of 50 scans covering 30° × 10° field size. BCVA and various SD-OCT parameters were collected every year and followed for up to five years. Lens status (phakia or pseudophakia) at the baseline and the number of injections of aflibercept were identified based on medical records.

### Morphologic analysis

Central retinal thickness (CRT) was measured as the distance between the inner border of the internal limiting membrane (ILM) and the inner border of the RPE. Subfoveal choroidal thickness (SFCT) was measured using enhanced depth scanning images as the distance between the outer surface of Bruch’s membrane and the chorioscleral interface. The presence of the foveal external limiting membrane (ELM) and ellipsoid zone (EZ) was evaluated using vertical and horizontal scans.

The presence of SRH was evaluated using color fundus photography. The area of SRH, the maximum height of SRH and hemorrhage extending beyond the ELM into the neurosensory retina were measured using color fundus photography and SD-OCT raster scans. The boundaries of SRH were determined based on hemorrhagic regions identified on color fundus images in conjunction with corresponding hyperreflective areas observed on SD-OCT. Distinction from fibrosis and pigmentary changes was made by integrating findings from both color fundus photography and SD-OCT. All assessments were performed independently by two graders (AY and YN), and inter-grader agreement was evaluated and found to be high (95.3%, 41/43 cases). Discrepancies were resolved by a retina specialist (MH). Retinal layer thickness was obtained using the automated segmentation function of Spectralis OCT (Supplementary Fig. 1A). All OCT images were carefully reviewed for segmentation accuracy, and manual corrections were performed when necessary^22^. Measurements were primarily conducted by a single masked grader (AY), and cases with potential segmentation uncertainty were further reviewed and confirmed by an experienced retinal specialist (MH). Retinal thickness was analyzed in three compartments: ganglion cell complex (GCC), inner nuclear layer (INL)-outer plexiform layer (OPL) complex, and ONL. GCC consisted of the retinal nerve fiber layer (RNFL), ganglion cell layer (GCL), and inner plexiform layer (IPL). The mapped 3 mm diameter area was divided into five sectors (superior, inferior, temporal, nasal and central), and the thickness of each layer was measured in each sector (Supplementary Fig. 1B). The center sector had a diameter of 1 mm. In each sector where hemorrhage was present, the absolute number of cases was also counted (Supplementary Fig. 1C).

We evaluated the presence of polypoidal lesions using ICGA at baseline in each case. Polypoidal choroidal vasculopathy (PCV) was diagnosed based on the findings of characteristic polypoidal lesions at the border of the branching choroidal vascular networks. Retinal angiomatous proliferation (RAP) was diagnosed by neovascularization associated with retinochoroidal anastomoses. Other cases were diagnosed with typical AMD (tAMD).

### Statistical analysis

Data are presented as means ± standard deviations where applicable. The BCVA was converted to the logarithm of the minimum angle of resolution (logMAR) for statistical analyses. Normality was assessed using the Shapiro–Wilk test. Comparative analyses between the two paired groups were performed using the paired t-test. Comparative analyses between the two independent groups were performed using the unpaired t-test for experimental data and the Mann–Whitney U test for clinical data, as appropriate. Categorical data were compared using the McNemar’s test for paired groups and the chi-square test for independent groups. Correlation analyses were performed using Spearman’s rank correlation coefficient. Multivariable analyses of one-year BCVA as the dependent variable were performed using linear regression analysis. All statistical analyses were performed using GraphPad Prism version 10 (GraphPad Software, LLC, San Diego, CA, USA) and R version 4.4.1 (R Foundation for Statistical Computing, Vienna, Austria). *P*-values < 0.05 were considered statistically significant.

## Results

**Fig. 2:**
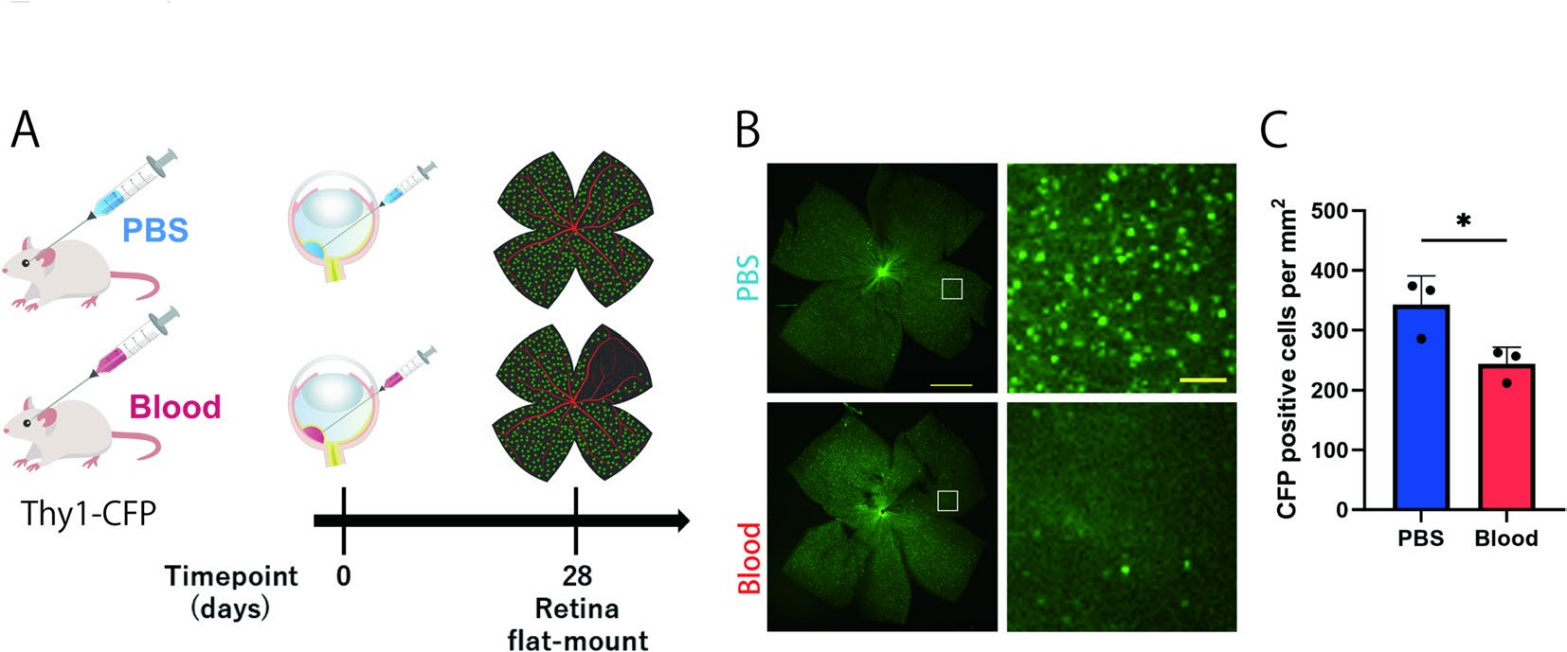
**(A)** Schematic illustration of the experimental timeline for retinal flat-mount analysis. **(B)** Representative retinal flat-mount images from Thy1-CFP mice at 28 days after subretinal injection of phosphate-buffered saline (PBS) or blood, showing reduced CFP-positive retinal ganglion cells (RGCs) in blood-injected eyes. Higher-magnification views of the boxed areas are shown in the right panels. Green fluorescent cells represent RGCs. Scale bars, 1000 µm (left panels) and 100 µm (right panels). **(C)** Quantification of CFP-positive RGC density (cells/mm^2^) in retinal flat-mounts demonstrating a significant reduction in blood-injected eyes compared with PBS-injected control eyes (n = 3 eyes from 3 independent mice per group). Unpaired t tests were used; *P < 0.05; error bars represent mean ± SEM.

Although SRH is known to damage photoreceptors in the outer retina and impair visual function, microglial activation has also been reported in the inner retina of eyes with SRH, suggesting that SRH may affect multiple retinal layers^20^. To investigate the effects of SRH on each retinal layer, we employed a mouse model of SRH by injection of autologous blood into the subretinal space. H&E staining revealed no apparent structural changes in the neurosensory retina at 48 and 96 h after blood injection; however, marked thinning of ONL was observed at 168 h (Fig. 1B). We found that the ONL was significantly thinner in the blood-injected eyes than in the PBS-injected control eyes at 168 h (Fig. 1C). TUNEL staining at 24 h after blood injection showed apoptotic cells predominantly in the ONL. At 48 h, an increased number of apoptotic cells was observed not only in the ONL but also in the GCL and INL compared with PBS-injected control eyes (Fig. 1, D and E), suggesting that apoptotic cell death may initially occur in the outer retina and subsequently extend to the inner retina. To evaluate the long-term effects of SRH on RGCs, we created an SRH model using Thy1-CFP transgenic mice, in which RGCs are specifically labeled with CFP (Fig. 2A). In retinal flat mounts obtained 28 days after subretinal injection of blood or PBS, the number of surviving RGCs was significantly lower in blood-injected eyes compared with PBS-injected eyes (Fig. 2B and C). Collectively, SRH affects not only photoreceptors adjacent to the hemorrhage but also distant retinal neurons, including retinal ganglion cells, indicating that SRH-induced injury spreads vertically across retinal layers in this model.

We next analyzed clinical data to determine whether SRH affects both the outer and inner retina in patients with neovascular AMD. First, we analyzed 43 eyes from 43 neovascular AMD patients (24 men and 19 women) who met the eligibility criteria. The mean age of the patients was 73.5 ± 8.5 years, and the mean BCVA (logMAR) was 0.25 ± 0.26. Among the 43 eyes, 16 had tAMD and 27 had PCV. All patients received 7.0 ± 0.0 injections of aflibercept during the first year (Supplementary Fig. 2A). In all eyes, mean BCVA was significantly improved from 0.25 ± 0.26 logMAR at baseline to 0.12 ± 0.28 logMAR at 1 year (*P* < 0.0001) (Supplementary Table 1). CRT and SFCT were significantly decreased (both *P* < 0.0001).

Among 43 eyes, 20 eyes showed SRH at baseline (SRH [ +] group) and 23 eyes did not (SRH [–] group) (Table 1). The mean area of SRH was 4.74 ± 8.88 mm^2^ (range: 0.46 – 37.69 mm^2^) and the mean maximum height of SRH was 267.5 ± 241.5 µm (range: 83.0 – 972.0 µm). SRH was more frequently observed in the central region (Supplementary Fig. 1C). Comparative analyses of baseline characteristics between the SRH ( +) group and the SRH (–) group revealed that the SRH ( +) group exhibited significantly worse BCVA (0.35 ± 0.33 logMAR vs 0.16 ± 0.13 logMAR, *P* = 0.047) and greater CRT (396.4 ± 178.2 µm vs 253.1 ± 98.7 µm, *P* = 0.003) than the SRH (–) group.

**Table 1:** Baseline characteristics of patients with neovascular AMD.

	Total	SRH (-) group	SRH ( +) group	*P*-value
Number of eyes, n	43	23	20	−
Age, years	73.5 ± 8.5	73.1 ± 7.0	74.0 ± 10.2	0.47
Sex (men/women), n	24/19	13/10	11/9	0.92
Axial length, mm	23.40 ± 0.88	23.46 ± 0.82	23.35 ± 0.96	0.62
Intraocular pressure, mmHg	12.6 ± 3.2	12.7 ± 3.1	12.6 ± 3.3	0.96
Lens status (phakia/IOL), n	37/6	20/3	17/3	0.85
BCVA, logMAR	0.25 ± 0.26	0.16 ± 0.13	0.35 ± 0.33	0.047*
Subtype (tAMD/PCV), n	16/27	11/12	5/15	0.12
CRT, μm	319.7 ± 157.2	253.1 ± 98.7	396.4 ± 178.2	0.003*
SFCT, μm	254.3 ± 95.4	248.9 ± 101.4	260.5 ± 90.1	0.67
Intact foveal EZ, % (+ /-, n)	60.5 (26/17)	73.9 (17/6)	45.0 (9/11)	0.05
Intact foveal ELM, % (+ /-, n)	72.1 (31/12)	87.0 (20/3)	55.0 (11/9)	0.02*
SRH area, mm^2^	–	–	4.74 ± 8.88	–
SRH height, μm	–	–	267.5 ± 241.5	–
Hemorrhage extending beyond the ELM, n	–	–	10	–

Data are presented as means ± standard deviations where applicable.

AMD = age-related macular degeneration; IOL = intraocular lens; BCVA = best corrected visual acuity; logMAR = logarithm of the minimum angle of resolution; tAMD = typical age-related macular degeneration; PCV = polypoidal choroidal vasculopathy; CRT = central retinal thickness; SFCT = subfoveal choroidal thickness, EZ = ellipsoid zone; ELM = external limiting membrane; SRH = subretinal hemorrhage.

* Statistically significant (*P* < 0.05).

**Fig. 3:**
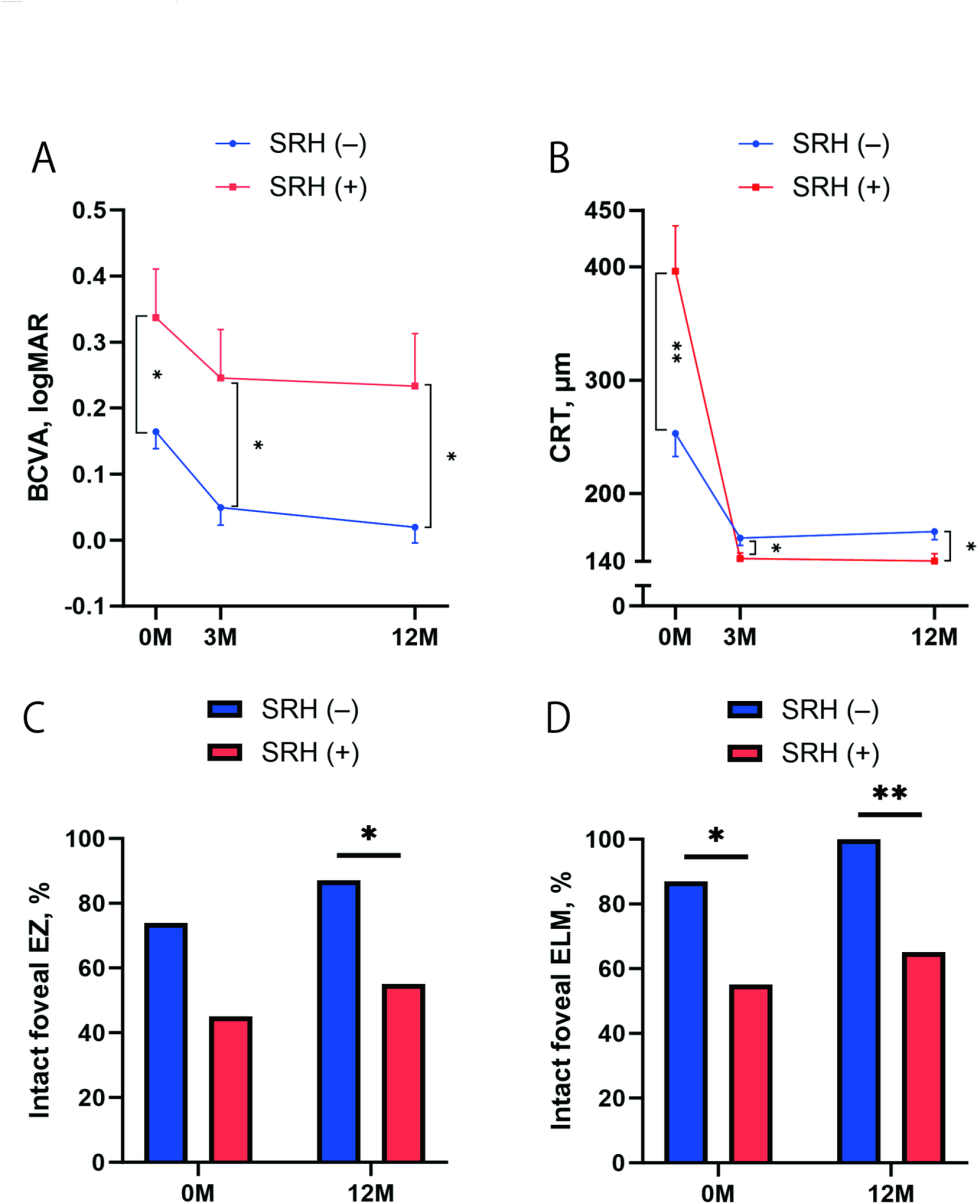
**(A)** Best-corrected visual acuity (BCVA, logMAR) at baseline (0 M), 3 months (3 M), and 12 months (12 M) in eyes without subretinal hemorrhage (SRH [–] group) and with SRH (SRH [ +] group). The SRH ( +) group showed significantly worse BCVA at all time points compared with the SRH (–) group. **(B)** Central retinal thickness (CRT, µm) at 0 M, 3 M, and 12 M in SRH (–) and SRH ( +) groups. CRT decreased markedly at 3 months in both groups and remained stable thereafter. **(C)** Proportion of eyes with intact foveal ellipsoid zone (EZ) at baseline and 12 months. **(D)** Proportion of eyes with intact foveal external limiting membrane (ELM) at baseline and 12 months. Mann–Whitney U tests (A and B) and McNemar’s test (C and D) were used; *P < 0.05, **P < 0.01; error bars represent mean ± SEM.

The SRH ( +) group exhibited significantly worse BCVA than the SRH (–) group at 3 months (0.25 ± 0.33 logMAR vs 0.05 ± 0.13 logMAR, *P* = 0.04) and at 1 year (0.23 ± 0.36 logMAR vs 0.02 ± 0.11 logMAR, *P* = 0.03) (Fig. 3A). Multivariable linear regression analysis revealed that baseline SRH was independently associated with poorer BCVA at 1 year (β = 0.16, 95% CI: 0.01–0.31, *P* = 0.03) (Supplementary Table 2). In the SRH ( +) group, CRT was significantly lower than the SRH (–) group at 3 months and at 1 year (142.6 ± 22.1 µm vs 158.4 ± 30.3 µm [*P* = 0.04], and 140.3 ± 29.6 µm vs 166.2 ± 33.7 µm [*P* = 0.02]) (Fig. 3B). In the SRH ( +) group, the rate of EZ disruption at baseline tended to be higher than that in the SRH (–) group, whereas the rate of ELM disruption was significantly higher (Fig. 3, C and D). At 1 year, the rates of both EZ and ELM disruption remained significantly higher in the SRH ( +) group compared with the SRH (–) group, and improvement in these disruptions tended to be less pronounced.

**Fig. 4:**
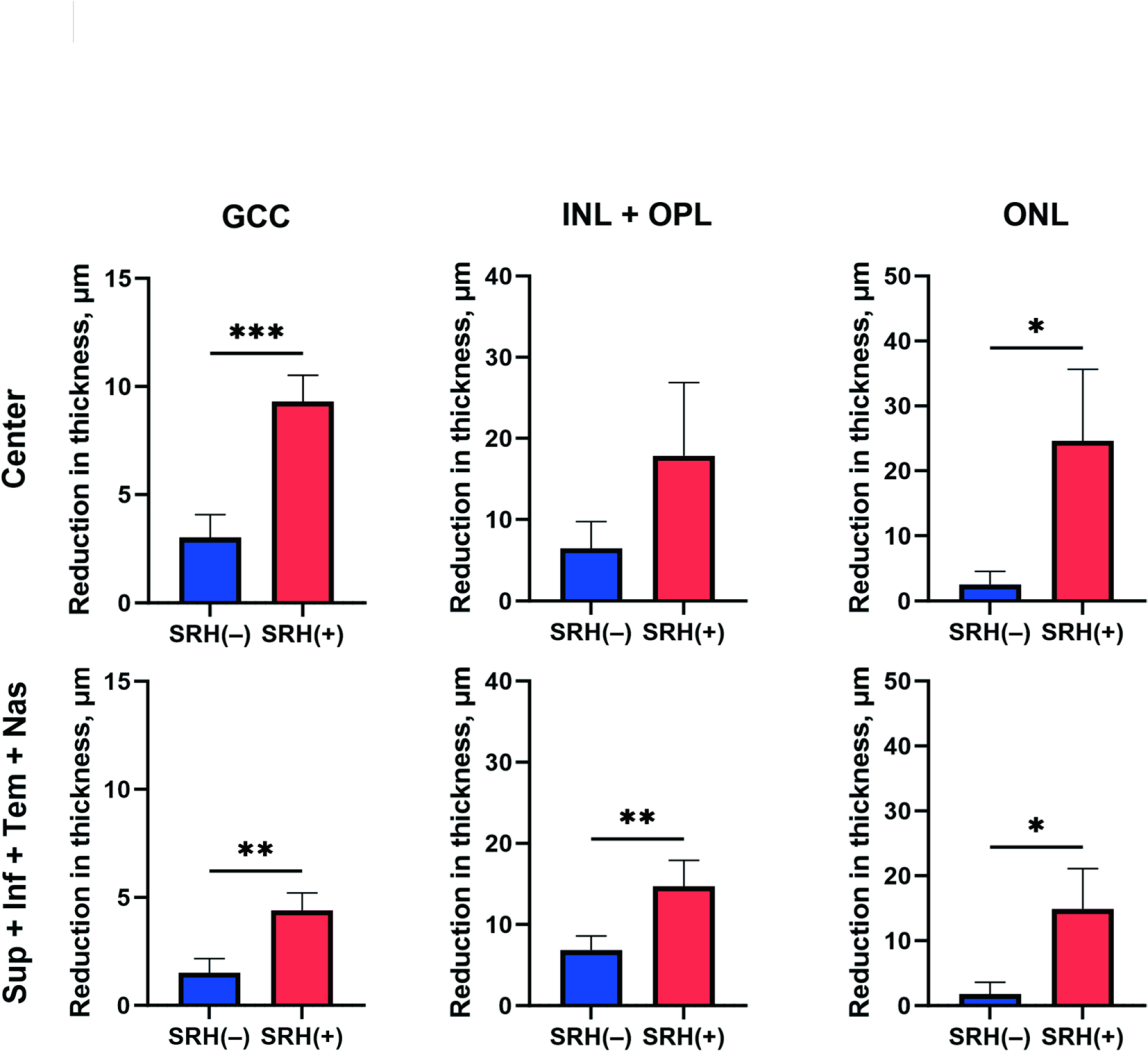
Reduction in retinal layer thickness at 1 year in the eyes without subretinal hemorrhage (SRH [–] group) and eyes with SRH (SRH [ +] group). Measurements were obtained separately for the central region (upper panels) and for the combined peripheral regions (superior + inferior + temporal + nasal; lower panels). Mann–Whitney U test was used; *P < 0.05, **P < 0.01, ***P < 0.001; error bars represent mean ± SEM.

We next focused on morphologic changes in each retinal layer and sector to clarify the structural impact of SRH, given that baseline SRH was associated with sustained impairment of visual outcomes and persistent disruption of retinal structure. Morphological analysis revealed that GCC and ONL thickness were significantly reduced in both the central and the average of four peripheral sectors in the SRH ( +) group (Fig. 4). Thinning of the INL + OPL complex was observed in the average of four sectors in the SRH ( +) group. Details of the reduction in the four sectors are provided in Supplementary Fig. 3.

**Fig. 5:**
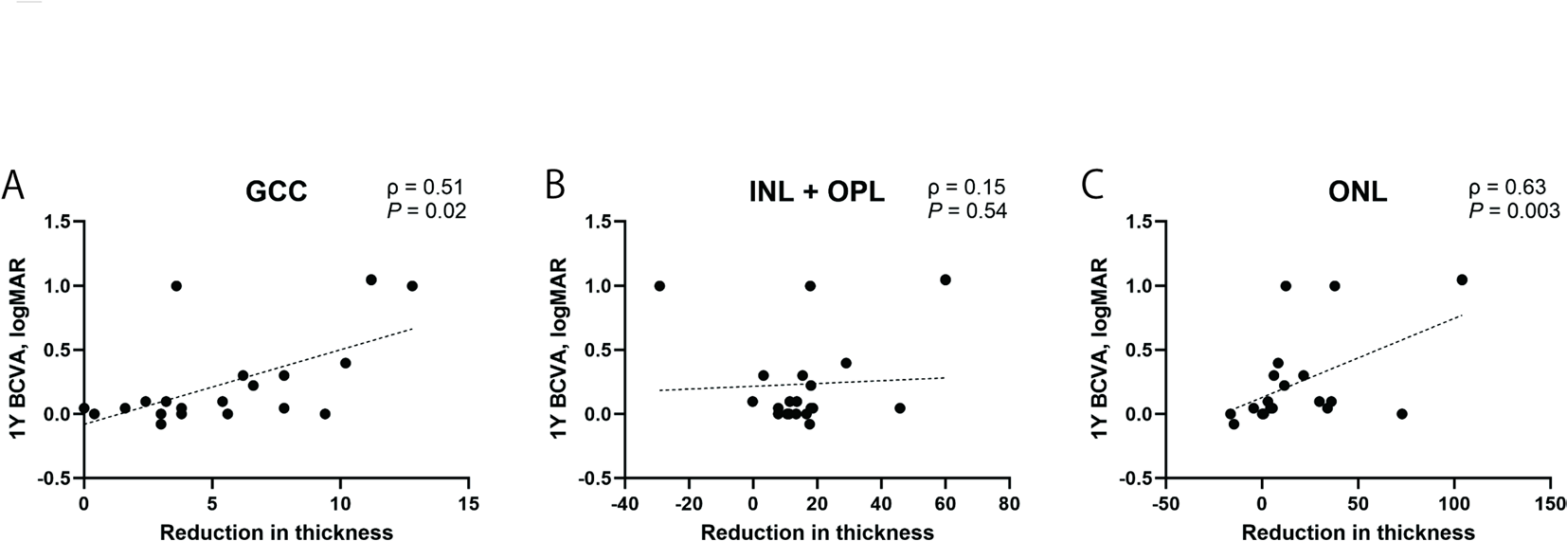
Scatter plots showing the relationship between the reduction in retinal layer thickness and best-corrected visual acuity at 1 year (1Y BCVA, logMAR). **(A)** Reduction in thickness of the ganglion cell complex (GCC). **(B)** Reduction in thickness of the inner nuclear layer and outer plexiform layer (INL + OPL). **(C)** Reduction in thickness of the outer nuclear layer (ONL). Dotted lines indicate linear regression lines. Correlations were assessed using Spearman’s rank correlation coefficient.

To clarify the relationship between retinal thinning and visual prognosis, we assessed correlations between 1-year BCVA and reduction in retinal layer thickness in the SRH ( +) group. 1-year BCVA was significantly correlated with reduction in thickness of GCC (ρ = 0.51, *P* = 0.02) and ONL (ρ = 0.63, *P* = 0.003) (Fig. 5, A and C). We investigated the associations between the reduction in thickness of the GCC and ONL and baseline characteristics (Table 2). The reduction in GCC and ONL thickness was associated with retinal infiltration of hemorrhage, CME, EZ disruption, and ELM disruption. In addition, the reduction in GCC thickness was also correlated with the height of SRH.

**Table 2:** Correlation between reduction in thickness and baseline clinical parameters.

	GCC		ONL	
	ρ	*P*	ρ	*P*
CRT	− 0.04	0.88	0.07	0.77
SFCT	0.08	0.74	− 0.23	0.34
SRH area	0.28	0.24	0.42	0.06
SRH height	0.47	0.04*	0.32	0.17
Retinal infiltration of hemorrhage	0.68	0.001*	0.64	0.002*
CME	0.56	0.01*	0.52	0.02*
EZ disruption	0.58	0.007*	0.76	0.0001*
ELM disruption	0.64	0.003*	0.62	0.004*

GCC = ganglion cell complex; ONL = outer nuclear layer; CRT = central retinal thickness; SFCT = subfoveal choroidal thickness; SRH = subretinal hemorrhage; CME = cystoid macular edema; EZ = ellipsoid zone; ELM = external limiting membrane.

* Statistically significant (P < 0.05).

We then focused on reduction in retinal thickness after the first year of follow-up to evaluate the long-term effects of SRH. Because the number of patients decreased over time, the longitudinal changes in sample size are provided in Supplementary Fig. 2B. In the SRH ( +) group, thickness reduction of the GCC and ONL was greater than the SRH (–) group during the first year (Supplementary Fig. 4). However, after the first year, the rate of thinning decreased, and reductions in retinal thickness were comparable between the SRH ( +) and SRH (–) groups.

## Discussion

This study demonstrated the impact of SRH on diverse neuronal populations across the entire retina in neovascular AMD. Our clinical data indicate that SRH causes not only ONL thinning but also GCC thinning, both of which were associated with poor visual prognosis despite intensive anti-VEGF treatment. Using an experimental SRH model generated by subretinal injection of autologous blood, we found that SRH induced cell death in multiple types of retinal neurons. These findings suggest that the detrimental effects of SRH are not limited to photoreceptors but extend to broader retinal structures. To the best of our knowledge, this is the first study to focus on neuronal cell death in the inner retinal layers and to investigate long-term retinal structural changes according to the presence or absence of SRH.

In this study, apoptotic cells were observed not only in the outer retina but also across all retinal layers, including the inner retina in the SRH model. These findings suggest that SRH-induced damage is not limited to local mechanical effects or direct contact with the blood clot but may propagate transversely across retinal layers. Notably, the temporal pattern observed in our study, with early involvement of the outer retina followed by subsequent extension to the inner retinal layers, suggests a combination of primary and secondary injury mechanisms. In the early phase, direct toxicity of blood components, including oxidative stress and iron-mediated damage, may predominantly affect photoreceptors^16–19^. In addition, mechanical stress caused by subretinal blood accumulation, such as compression and clot retraction, may contribute to structural disruption^14,15^. Previous studies have reported that in experimental SRH models, microglial activation has been observed not only at the hemorrhage site but also in adjacent inner retinal regions without direct contact with the blood clot, implying the involvement of diffusible blood-derived or inflammatory factors^20,23^. Consistent with these observations, in the present study, apoptotic cells were detected throughout the retina, and RGC loss was also observed even at a long-term time point of 28 days. These findings suggest that an SRH-induced inflammatory environment may contribute to neuronal damage not only in the outer retina but also within the inner retinal layers.

Previous clinical studies have reported that SRH is associated with severe retinal structural damage, resulting in poor visual prognosis^4,7,24^. Our findings are consistent with these reports. Patients with baseline SRH exhibited significantly worse visual acuity than those without SRH, not only at baseline but also after the loading phase of aflibercept treatment and at 1 year. Importantly, the persistence of visual impairment despite anti-VEGF therapy suggests that the impact of SRH is not merely transient or secondary to exudative activity. In the SRH ( +) group, increased baseline CRT likely reflects acute exudation and hemorrhage-related retinal swelling. Following anti-VEGF treatment, resolution of exudative changes led to a reduction in retinal thickness. However, the subsequent thinning observed at later time points may not represent simple anatomical improvement, but rather reflect structural damage such as retinal atrophy or fibrosis secondary to SRH, potentially involving multilayer retinal damage. Furthermore, multivariable analysis demonstrated that SRH was independently associated with visual outcomes at 1 year after adjustment for other clinical factors, indicating that SRH itself may contribute to long-term visual dysfunction. These findings underscore the importance of elucidating the structural and biological mechanisms by which SRH induces sustained retinal damage.

Morphological analysis revealed that SRH induced thinning across multiple retinal layers, particularly the GCC and ONL. Thinning of both the GCC and ONL showed a significant negative correlation with visual acuity at 1 year. The retina contains first-, second- and third-order neurons, with the ONL comprising photoreceptors, the first-order neurons of the visual pathway^25^. The observed association between ONL thinning and visual acuity suggests that photoreceptor damage plays a critical role in visual dysfunction in eyes with SRH. Notably, GCC thinning was also associated with visual prognosis. Because the GCC includes retinal ganglion cells, which correspond to the third-order neurons of the visual pathway, impairment of this layer may adversely affect visual functions such as visual field sensitivity and contrast sensitivity^26–29^. Previous reports have shown that the height and area of hemorrhage are associated with final visual acuity^30^ and in our analyses, the extent of SRH was significantly correlated with GCC thinning, whereas SRH height tended to be associated with ONL thinning. Collectively, these findings suggest that greater SRH burden may lead to more extensive neuronal damage across retinal layers, thereby resulting in worse visual outcomes.

This study had several limitations. First, the clinical component was a single-center retrospective study with a relatively small number of cases, and some patients were lost to follow-up during the long-term observation period. In addition, differences in disease subtype (e.g., PCV vs typical AMD) may have influenced retinal morphology and visual outcomes. Future prospective studies with larger cohorts are warranted to validate our findings. Second, although we performed detailed morphologic analyses of retinal structure, functional assessment was limited to visual acuity. Because GCC thinning may reflect inner retinal neuronal dysfunction, additional functional evaluations such as visual field testing or microperimetry would provide a more comprehensive understanding of the clinical significance of inner retinal damage. Third, while our animal experiments demonstrated multilayer retinal cell death, the precise molecular mechanisms and potential therapeutic interventions were beyond the scope of this study and should be addressed in future investigations.

In conclusion, our study demonstrated that baseline SRH is an independent predictor of poor visual outcomes in patients with neovascular AMD. SRH induced thinning across multiple retinal layers, with GCC and ONL thinning showing strong associations with visual dysfunction. We also established an in vivo SRH model by subretinal injection of blood, which recapitulated both photoreceptor and ganglion cell death and provided mechanistic support for the clinical findings. Future studies integrating detailed functional assessments with experimental approaches may further elucidate the mechanisms of SRH-induced retinal injury and facilitate the development of therapeutic strategies aimed at protecting retinal neurons.

## Supplementary Information

Below is the link to the electronic supplementary material.

Supplementary Material 1

Supplementary Material 2

## Data Availability

The datasets generated during and/or analyzed during the current study are available from the corresponding author on reasonable request.
