# Supplementary material for: Experimental and clinical evidence of multilayer retinal damage caused by subretinal hemorrhage in neovascular age-related macular degeneration
Source: Sci Rep. 2026 May 13;16:21909. doi: 10.1038/s41598-026-52680-8 (PMC13365463; doi:10.1038/s41598-026-52680-8)

Supplementary Figure 1

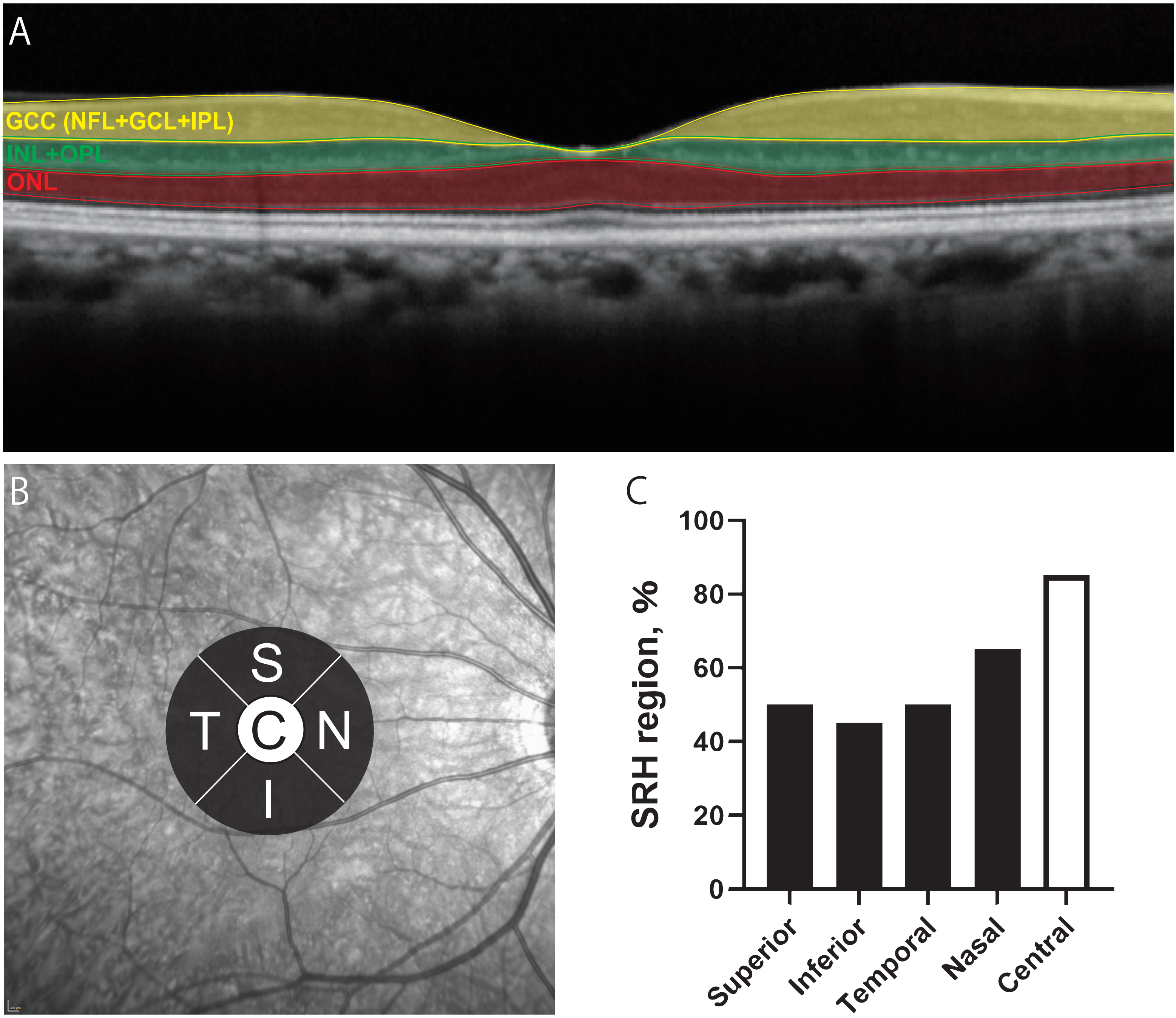

Supplementary Figure 2

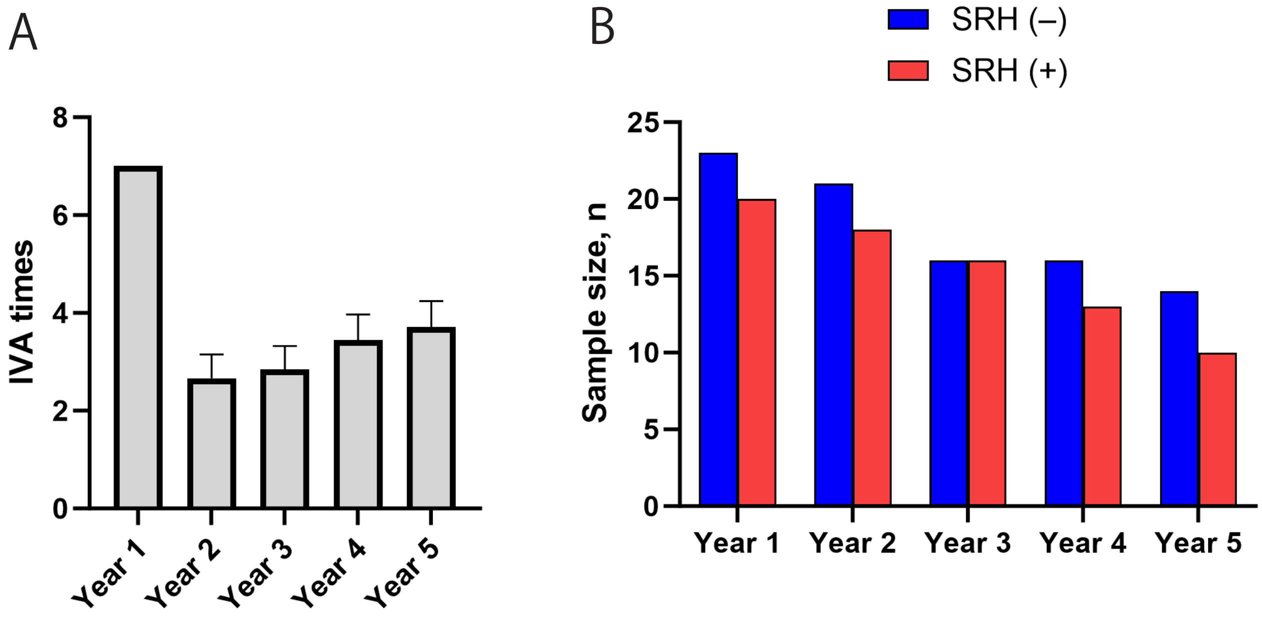

Supplementary Figure 3

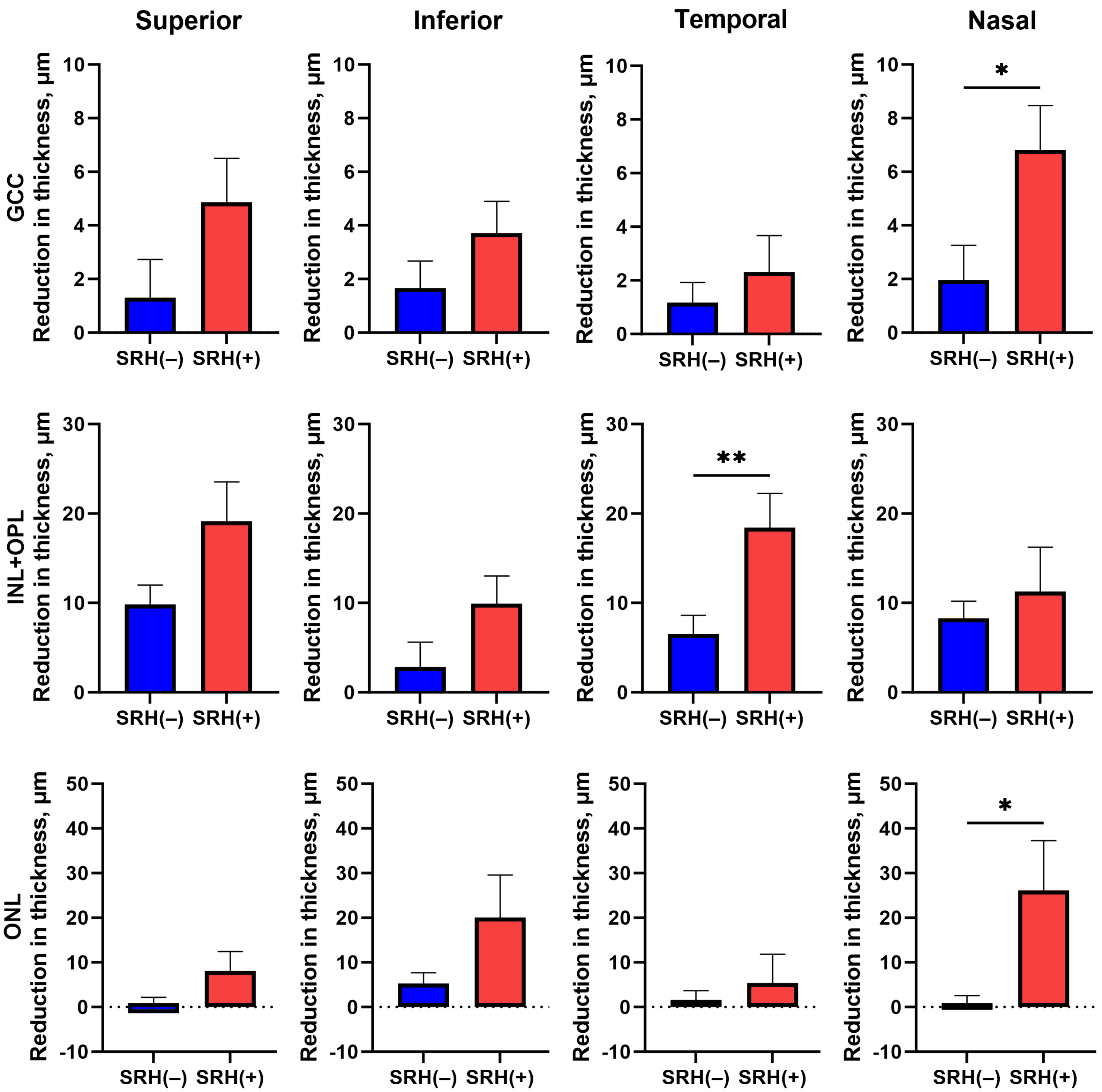

Supplementary Figure 4

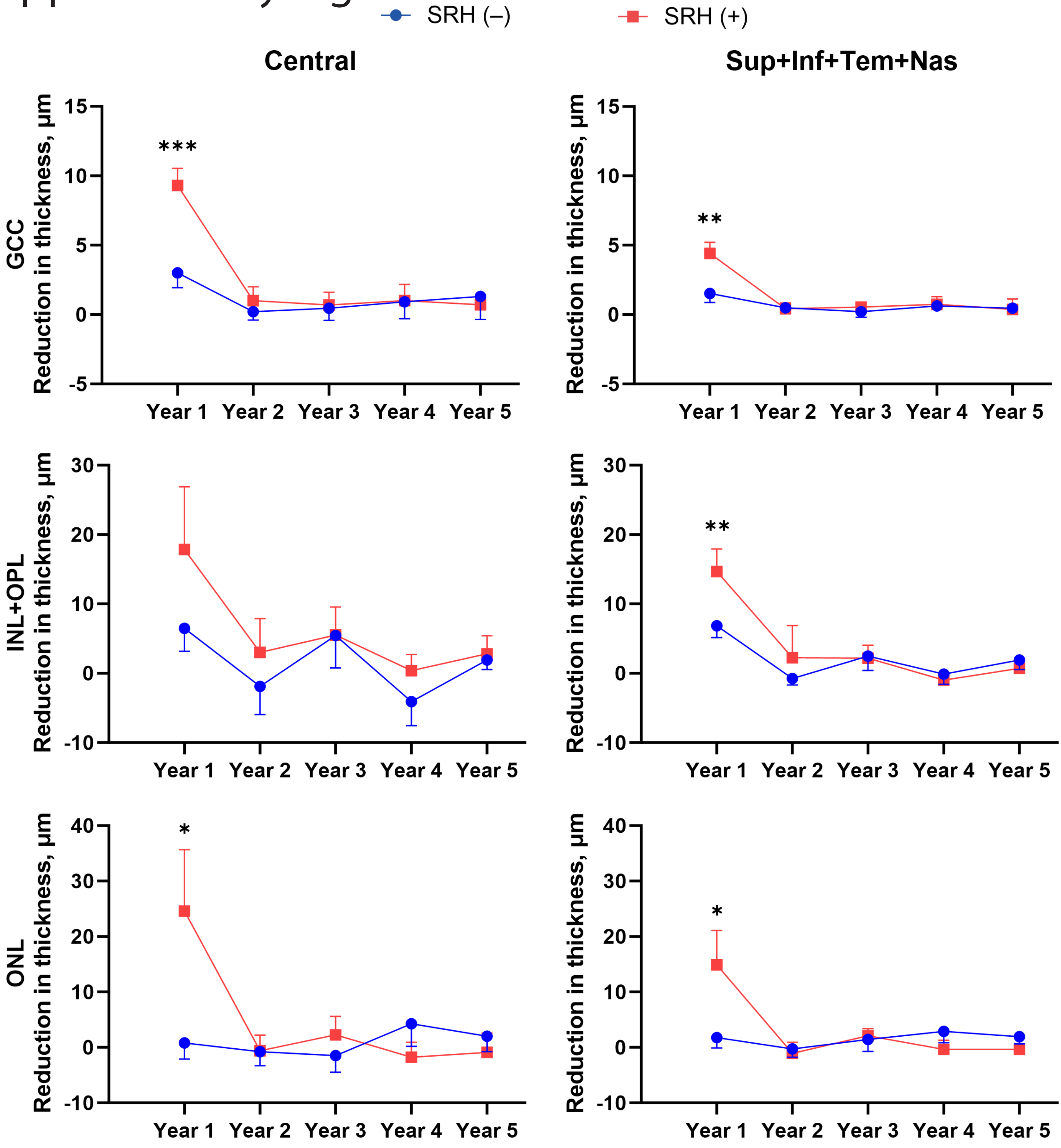

Supplement: Supplementary file 1 — Supplementary Material 1 [file 41598_2026_52680_MOESM1_ESM.pdf]
