# Supplementary material for: Experimental and clinical evidence of multilayer retinal damage caused by subretinal hemorrhage in neovascular age-related macular degeneration
Source: Sci Rep. 2026 May 13;16:21909. doi: 10.1038/s41598-026-52680-8 (PMC13365463; doi:10.1038/s41598-026-52680-8)
Supplement: Supplementary file 2 — Supplementary Material 2 [file 41598_2026_52680_MOESM2_ESM.pdf]

**Supplementary Table 1. Comparison of baseline and 1-year characteristics in patients with neovascular AMD**

| | Baseline | 1-year | *P*-value |
|---|---|---|---|
| BCVA, logMAR | 0.25 ± 0.26 | 0.12 ± 0.28 | <0.0001* |
| CRT, μm | 319.7 ± 157.2 | 154.1 ± 34.1 | <0.0001* |
| SFCT, μm | 254.3 ± 95.4 | 226.7 ± 88.8 | <0.0001* |
| Intact foveal EZ, % (+/－, n) | 60.5 (26/17) | 74.4 (32/11) | 0.23 |
| Intact foveal ELM, % (+/－, n) | 72.1 (31/12) | 86.0 (37/6) | 0.13 |

Data are presented as means ± standard deviations where applicable.
AMD = age-related macular degeneration; BCVA = best corrected visual acuity; logMAR = logarithm of the minimum angle of resolution; CRT = central retinal thickness; SFCT = subfoveal choroidal thickness, EZ = ellipsoid zone; ELM = external limiting membrane
* Statistically significant (*P* <0.05)

**Supplementary Table 2. Correlation between 1-year BCVA and baseline clinical parameters**

| | Univariable analysis | | Multivariable analysis | |
|---|---|---|---|---|
| | ρ | *P* | *β* (95% CI) | *P* |
| Age | 0.44 | 0.003* | 0.01 (0.003 to 0.02) | 0.01* |
| Sex | 0.02 | 0.90 | − | − |
| CRT | −0.02 | 0.89 | − | − |
| SFCT | −0.16 | 0.31 | − | − |
| SRH | 0.34 | 0.03* | 0.16 (0.01 to 0.31) | 0.03* |
| EZ | 0.27 | 0.08 | 0.14 (−0.02 to 0.30) | 0.08 |
| ELM | 0.20 | 0.19 | − | − |

BCVA = best corrected visual acuity; CI = confidence interval; CRT = central retinal thickness; SFCT = subfoveal choroidal thickness; SRH = subretinal hemorrhage; EZ = ellipsoid zone; ELM = external limiting membrane

* Statistically significant ($P$ <0.05)
